# Giant Renal Artery Aneurysms: Decision-Making Dilemmas

**DOI:** 10.1155/2013/390928

**Published:** 2013-09-10

**Authors:** Michael J. Ramdass

**Affiliations:** Department of Surgery, General Hospital, Charlotte Street, Port-of-Spain, Trinidad and Tobago

## Abstract

A case of a 10 cm giant renal artery aneurysm in an 81-year-old lady is herein reported. The patient presented as an incidental finding on an abdominal ultrasound with an aneurysm which was large, soft, egg-shell-like, and bilobular with a cauliflower-type pattern invading the parenchyma of the renal substance and extending caudally. A right nephrectomy was carried out, and the patient made an uneventful recovery. This paper reviews the options for treatment of giant renal artery aneurysms and highlights the benefit of a planned nephrectomy once there is adequate renal reserve in the opposite kidney using a midline approach for right-sided giant renal artery aneurysms and a left-sided flank approach for left-sided aneurysms.

## 1. Introduction

The first published report of a renal artery aneurysm (RAA) was in 1770 by Rouppe, who described the demise of a sailor who fell onto his right flank. Autopsy revealed a large false aneurysm with rupture [1]. Since then, the literature has been filled with a few case reports on giant renal artery aneurysms explaining the pathology in various situations and management options. This paper focuses on the presentation and management of one of the largest giant renal artery aneurysms worldwide and summarises the literature with a proposal on treatment options of this rare and hazardous condition.

## 2. Case Report

An 81-year-old lady of African descent was referred from a peripheral centre with right upper quadrant discomfort and hypertension. She was thought to have gallstones with biliary colic from the history. Investigation with ultrasound revealed a normal gallbladder and a giant renal artery aneurysm measuring approximately 10 cm involving the renal parenchyma. Her hypertension was controlled with nifedipine.

**Figure 1:**
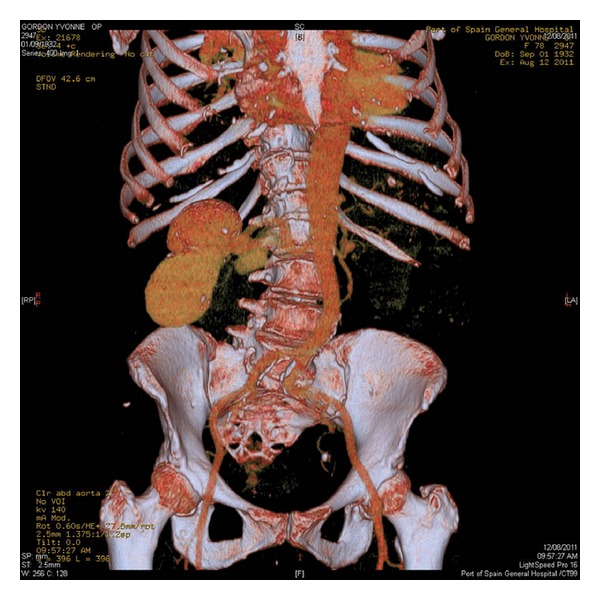
CT angiogram reconstruction showing a complex bilobular right renal artery aneurysm of approximately 10 cm wider diameter and a smaller aneurysm of 3 cm involving the distal renal artery and extending into the renal parenchyma.

A CT angiogram of the aorta and renal arteries showed a complex multiloculated aneurysm of the right renal artery extending into the renal parenchyma and involving the distal renal artery. The widest measurement was approximately 10 cm with a smaller component of approximately 3 cm (Figure 1).

**Figure 2:**
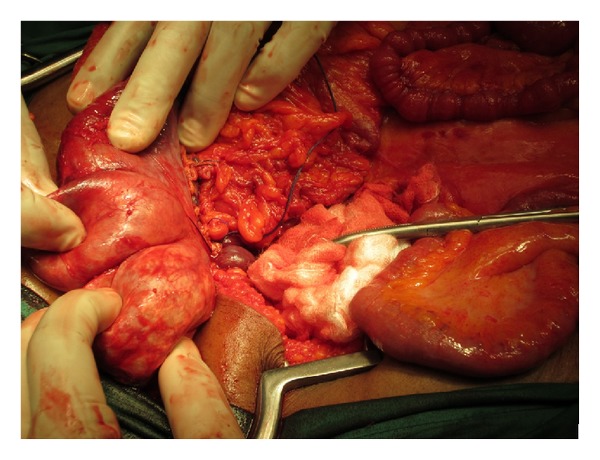
Intraoperative photo of the giant bilobular aneurysm with ligation of the renal artery flush with the aorta.

A renal scintigraphy showed the right kidney to be 10% functional and the left to be 80%. After careful deliberation on surgical options, the patient was advised and consented to have a right nephrectomy. A midline laparotomy approach was done in order to get full control of the right renal artery as it branched from the aorta. Proximal control was obtained and the renal artery and vein double ligated with 2.0 prolene early in the procedure. To note, the aneurysm was soft and felt like an eggshell that was easy to crack or injure accidentally; hence, the dissection was carried out gingerly (Figure 2).

**Figure 3:**
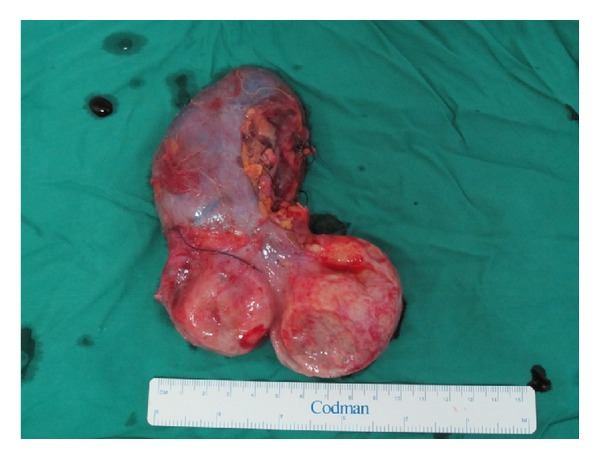
Photo showing the bilobular renal artery aneurysm.

**Figure 4:**
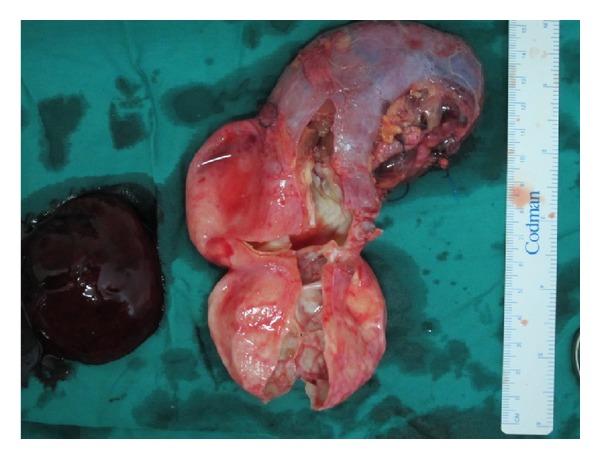
Photo showing the aneurysm with an eggshell-like appearance and cauliflower-type pattern of development, measuring approximately 10 cm in widest diameter.

The specimen looked like a bilobular cauliflower-type pattern in the parenchyma of the renal substance extending caudally. It was large with an extremely thin wall and very friable. There was a large fresh clot within the sac, which probably developed after early ligation of the renal artery flush with the aorta (Figures 3 and 4).

The patient had a smooth postoperative recovery and went home with no compromise in renal function and good control of her hypertension on nifedipine. 

## 3. Discussion

Renal artery aneurysm (RAA) is a rare entity with an incidence of less than 1% worldwide [2]. One of the largest series analysing RAAs was by Henke et al. published in the Annals of Surgery in 2001 where it was found that out of 168 patients (107 women, 61 men) there were 252 RAAs encountered over 35 years at the University of Michigan Hospital. They were solitary in 115 patients and multiple in 53 patients being bilateral in 32 cases. Disease associations included hypertension (73%), renal artery fibrodysplasia (34%), systemic atherosclerosis (25%), and extrarenal aneurysms (6.5%). They also found that most were saccular (79%) and noncalcified (63%) with the main renal artery bifurcation being the most common site of aneurysms (60%). Most RAAs were asymptomatic (55%), with a diagnosis being made during arteriography [3]. Other associations include vasculitis, congenital abnormalities, penetrating trauma or iatrogenic causes including biopsy or nephrostomy, infection, neurofibromatosis [4], and even dissection [5]. Complications include rupture, thrombosis, embolisation, hypertension, and arteriovenous fistula. However, most present as an incidental finding as in Henke et al.'s study [3] and likewise in the case herein presented. 

The association with renovascular hypertension is unexplained and possible explanations include anatomic kinking of the renal artery, segmental renal parenchymal ischaemia, flow turbulence, or coexistent renal artery stenosis [3].

Giant renal artery aneurysms (GRAA) are even rarer than the standard RAA with only a handful of case reports in the world literature offering explanations on management. Looking at the literature it seems as though that the giant renal artery aneurysms documented range in size from 5 to 12 cm [6–13]. There have been two more rare cases the first in a child and reported since 1982 [14] and another diagnosed postpartum [15] highlighting the point that these may be associated with pregnancy.

Henke et al. also very accurately described an algorithm for the treatment of RAAs whereby small unilateral RAAs are observed. Planned nephrectomy should be performed for complex disease which is not reconstructable or those with advanced parenchymal disease and lastly, planned arterial reconstructions may be performed in those with reconstructable disease. Planned arterial reconstructions included aneurysmectomy with bypass grafting, aneurysmectomy with primary angioplasty, or segmental renal artery implantation [3].

Another paper by English et al. describes surgical repair using cold perfusion preservation in situ and ex vivo techniques with no unplanned nephrectomies and good results overall [16]. Endovascular treatment via stenting or embolisation can also be performed for smaller, suitable RAAs with the risk of renal infarction, severe flank pain, and haematuria [17].

We advocate an open approach for GRAAs, depending on the anatomic arrangement of the vessels and the location of the aneurysm. Then either arterial reconstruction or planned nephrectomy can be performed. In this particular case, a safe approach is the transabdominal approach since the aneurysm was very large, friable, and located on the right side. This is in contrast to the technique described by Naraynsingh et al. in 2009 in his paper on a safe surgical approach to a giant intrarenal arteriovenous fistula and aneurysm [18] which was located on the left side and is much easier to approach through the flank as in a retroperitoneal abdominal aortic aneurysm repair. 

## 4. Conclusion

In conclusion, much has been written about the management of RAAs; however, little exists on treatment plans for giant RAAs. It seems that most Giant RAAs are detected when they are close to 10 cm in diameter and are usually part of the renal parenchyma due to their shear size. Arterial reconstruction is not always an option since these patients may be elderly and have significant comorbidities. This document advocates the view of planned nephrectomy once there is adequate renal reserve in the opposite kidney using a midline approach for right sided GRAAs and a left sided flank approach for left sided GRAAs.
